# Activation of mGluR5 Attenuates NMDA-Induced Neurotoxicity through Disruption of the NMDAR-PSD-95 Complex and Preservation of Mitochondrial Function in Differentiated PC12 Cells

**DOI:** 10.3390/ijms150610892

**Published:** 2014-06-17

**Authors:** Shu-Hui Dai, Na Qin, Tao Chen, Peng Luo, Lei Zhang, Wei Rao, Yue-Fan Yang, Xiao-Fan Jiang, Zhou Fei

**Affiliations:** 1 Department of Neurosurgery, Xijing Institute of Clinical Neuroscience, Xijing Hospital, Fourth Military Medical University, Xi’an 710032, China; E-Mails: fmmudaishuhui@163.com (S.-H.D.); fmmuchentao@163.com (T.C.); fmmuluopeng@163.com (P.L.); fmmuzhanglei@163.com (L.Z.); fmmuraowei@163.com (W.R.); fmmuyangyf@126.com (Y.-F.Y.); fmmujiangxiaofan@163.com (X.-F.J.); 2 Department of Neurology, Xijing Hospital, Fourth Military Medical University, Xi’an 710032, China; E-Mail: fmmuqinna@163.com; 3 Department of Surgery, the 123th Hospital of PLA, Bengbu 233000, China

**Keywords:** glutamate, mGlu receptor, NMDAR (*N*-methyl-d-aspartate receptor) receptor, post-synaptic density protein 95, mitochondrial dysfunction

## Abstract

Glutamate-mediated toxicity is implicated in various neuropathologic conditions, and activation of ionotropic and metabotropic glutamate receptors is considered to be the most important mechanism. It has been reported that pharmacological saturation of metabotropic glutamate receptors (mGluRs) can facilitate *N*-methyl-d-aspartate receptor (NMDAR) related signaling cascades, but the mechanism leading to mGluR-NMDAR interactions in excitotoxic neuronal injury has remained unidentified. In the present study, we investigated the role of mGluR5 in the regulation of *N*-methyl-d-aspartate (NMDA)-induced excitotoxicity in differentiated PC12 cells. We found that activation of mGluR5 with the specific agonist *R*,*S*-2-chloro-5-hydroxyphenylglycine (CHPG) increased cell viability and inhibited lactate dehydrogenase (LDH) release in a dose-dependent manner. CHPG also inhibited an increase in the Bax/Bcl-2 ratio, attenuated cleavage of caspase-9 and caspase-3, and reduced apoptotic cell death after NMDA treatment. The NMDA-induced mitochondrial dysfunction, as indicated by mitochondrial reactive oxygen species (ROS) generation, collapse of mitochondrial membrane potential (MMP), and cytochrome c release, was also partly prevented by CHPG treatment. Furthermore, CHPG blocked the NMDA-induced interaction of NMDAR with postsynaptic density protein-95 (PSD-95), but had no effects on intracellular calcium concentrations. All these results indicated that activation of mGluR5 protects differentiated PC12 cells from NMDA-induced neuronal excitotoxicity by disrupting NMDAR-PSD-95 interaction, which might be an ideal target for investigating therapeutic strategies in various neurological diseases where excitotoxicity may contribute to their pathology.

## 1. Introduction

Glutamate is the principal excitatory neurotransmitter in the central nervous system (CNS), modulating a series of physiological and pathophysiological processes [1]. Excessive release of glutamate can lead directly to excitotoxicity in neurons through both the ionotropic ligand-gated ion-channel ionotropic receptors (iGluRs), including the *N*-methyl-d-aspartate receptor (NMDAR) and α-amino-3-hydroxy-5-methyl-4-isoxazole propionate receptor (AMPAR), as well as G-protein-coupled metabotropic glutamate receptors (mGluRs). Glutamate and its receptors-mediated excitotoxicity is thought to play a role in many neurological diseases, such as brain trauma [2], stroke [3], Alzheimer’s disease [4], and Parkinson’s disease [5]. Multiple previous studies demonstrated an association between glutamate receptor function and scaffold proteins at the postsynaptic level. Postsynaptic density-95 (PSD-95), as a major protein constituent of these postsynaptic scaffold proteins, may play an important role by coupling glutamate receptors such as NMDAR and intracellular modulators [6,7,8,9]. The protein-protein interactions probably provide an important approach to facilitate glutamate receptor transmission and participate in the etiology of neurological diseases. It is reported that disturbing the NMDAR-PSD-95 interaction can reduce excitotoxic damage in experimental stroke models [10].

Other than the NMDAR, PSD-95 also functions as a scaffold to assemble a specific set of signaling proteins, such as neuronal nitric oxide synthase (nNOS), around the NMDAR into a macromolecular signaling complex. PSD-95, in turn, interacts with scaffold proteins lying in the deep part of the PSD, such as Shank, which interacts with the mGluRs through Homer. Although there have been many studies showing the potentiating effects of *N*-methyl-d-aspartate (NMDA) on mGluR5 function [11,12,13], the mechanisms responsible for mGluR modulation of NMDAR function still remain unclear. Thus, further elucidation of the interactions between these proteins in excitotoxicity can reveal alternative therapeutic targets for the treatment of neurological diseases. In this study, we explored the effects of mGluR5 modulation on apoptosis and mitochondrial function in differentiated PC12 cells after NMDA-induced neurotoxicity. We attempted to identify a potential mechanism for the inhibitory effect of mGluR5 activation on NMDAR. Furthermore, we provided evidence that this inhibition was partly due to disruption of the NMDAR-PSD-95 interaction.

## 2. Results and Disscussion

### 2.1. R,S-2-Chloro-5-hydroxyphenylglycine (CHPG) Protects against NMDA-Induced Neurotoxicity

**Figure 1:**
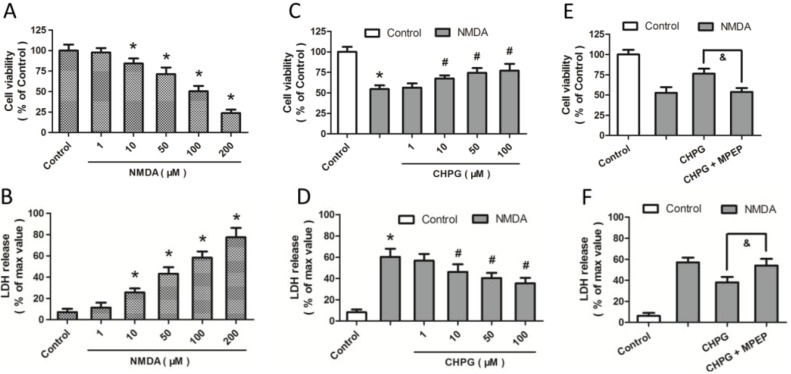
CHPG (*R*,*S*-2-chloro-5-hydroxyphenylglycine) protects against NMDA (*N*-methyl-d-aspartate)-induced neurotoxicity. Differentiated PC12 cells were treated with NMDA at different concentrations (1, 10, 50, 100 or 200 μM) for 24 h, and cell viability (**A**) and LDH (lactate dehydrogenase) release (**B**) were assayed, respectively; (**C**,**D**) Cells were pretreated with CHPG at varying doses (1, 10, 50 or 100 μM) 1 h before exposure to 100 μM NMDA for 24 h, and cell viability and LDH release were assayed subsequently; (**E**,**F**) Cells were pretreated with CHPG (50 μM), 1 h before NMDA treatment with or without 2-Methyl-6-(phenylethynyl)pyridine (MPEP) (20 μM), and cell viability and LDH release were assayed subsequently. Data are represented as means ± SD from six experiments. * *p* < 0.05 *vs.* control group and ^#^ *p* < 0.05 *vs.* cells treated only with NMDA. ^&^ *p* < 0.05.

To investigate the effects of NMDA on excitotoxicity-associated apoptosis, differentiated PC12 cells were incubated with various concentrations of NMDA from 1 to 200 μM for 24 h. The subsequent assessment of cell viability and LDH release indicated that NMDA caused concentration-dependent cell death (Figure 1A,B). Under our experimental conditions, approximately 75% or 50% of PC12 cells died after incubation for 24 h with 200 or 100 μM NMDA, respectively, whereas less than 50% of PC12 cells died after incubation for 24 h with 1, 10 or 50 μM NMDA. Thus, we confirmed NMDA-induced loss of cell viability in PC12 cells. To determine the biological functions of mGluR5 in NMDA-induced excitotoxicity, differentiated PC12 cells were pretreated with CHPG, an mGluR5 agonist, at different doses (1, 10, 50, 100 μM), 1 h before exposure to 100 μM NMDA for 24 h. An increase in cell viability and a decrease in LDH release indicated that CHPG, at a concentration of 10 μM or higher, prevented NMDA-induced neurotoxicity (Figure 1C,D). The cells were therefore treated with 100 μM NMDA or 50 μM CHPG for 24 h in subsequent experiments to induce an injury level of 50% and a significant protective effect. We used 2-Methyl-6-(phenylethynyl)pyridine (MPEP), a mGluR antagonist, to further confirm that the protective effect was due to mGluR5 activation. The results showed that the CHPG-induced increase in cell viability and decrease in LDH release after NMDA treatment were all fully prevented by MPEP pretreatment, indicating that the CHPG-induced protection against NMDA injury was dependent on mGluR5 activation.

### 2.2. CHPG Inhibits NMDA-Induced Apoptotic Cell Death

**Figure 2:**
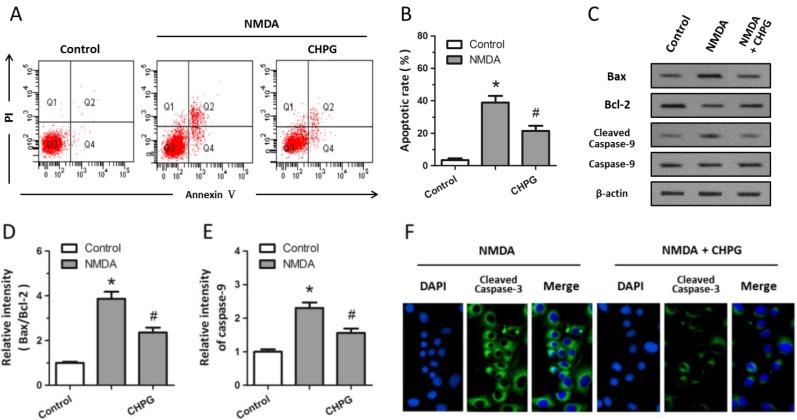
CHPG inhibits NMDA-induced apoptotic cell death. Differentiated PC12 cells were treated with 100 μM NMDA alone or pretreated with 50 μM CHPG before NMDA treatment. (**A**) Representative flow cytograms of annexin V-PI in different groups; and (**B**) mean cell fluorescence for viable cells (Annexin V^−^/PI^−^), early apoptotic (Annexin V^+^/PI^−^), and late apoptotic cells (Annexin V^+^/PI^+^), indicating the apoptotic rate of different groups; (**C**) Western blotting was performed to determine the expression of Bax, Bcl-2, caspase-9, and cleaved caspase-9 protein levels; The Bax/Bcl-2 ratio (**D**) and relative intensity of caspase-9 (**E**) were calculated; The cleavage of caspase-3 was measured by immunostaining (**F**). Data are represented as means ± SD from six experiments. * *p* < 0.05 *vs.* control group and ^#^ *p* < 0.05 *vs.* cells treated only with NMDA.

To further clarify the effect of CHPG on apoptotic cell death, annexin V-PI flowcytometry was performed, as shown by the representative cytograms in Figure 2A. Both necrotic cells in Q2 (Annexin V^+^/PI^+^) and apoptotic cells in Q4 (Annexin V^+^/PI^−^) were significantly increased after exposure to 100 μM NMDA for 24 h compared to the control group, whereas the necrosis index and quantification of the percentage of apoptotic PC12 cells showed a significant decrease when cells were pretreated with CHPG (Figure 2B). Western blot analysis was used to detect the expression of apoptosis-associated molecules (Figure 2C). The results showed that there was a significant increase in the Bax/Bcl-2 ratio when cells were treated with 100 μM NMDA, which also caused the activation of caspase-9, as indicated by the increased expression of cleaved caspase-9 (Figure 2D,E). Moreover, immunostaining indicated an increase in the cleavage of caspase-3 after NMDA treatment (Figure 2F). However, activation of mGluR5 by pretreatement with CHPG significantly attenuated the increase in the Bax/Bcl-2 ratio, the activation of caspase-9, and the cleavage of caspase-3, thereby demonstrating that pretreatment of CHPG protected PC12 cells from NMDA-induced apoptosis to some degree.

### 2.3. CHPG Attenuates NMDA-Induced Mitochondrial Dysfunction

**Figure 3:**
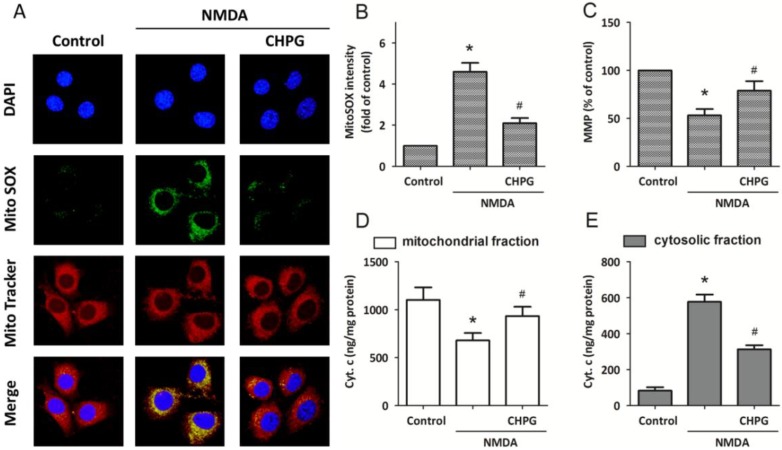
CHPG attenuates NMDA-induced mitochondrial dysfunction. Differentiated PC12 cells were treated with 100 μM NMDA alone or pretreated with 50 μM CHPG before NMDA treatment. (**A**) ROS (reactive oxygen species) generation in mitochondria was assessed by MitoSOX (green) and MitoTracker (red) staining, and MitoSOX intensity was calculated (**B**); The mitochondrial membrane potential (MMP) value was determined (**C**) and the expression levels of cytochrome c in mitochondrial fraction (**D**) and cytosolic fraction (**E**) were measured, respectively. Data representmeans ± SD from five experiments. * *p* < 0.05 *vs.* control group and ^#^ *p* < 0.05 *vs.* cells treated only with NMDA.

To determine whether CHPG induced mitochondrial dysfunction under NMDA-induced apoptotic cell death, MitoSOX was used to specifically quantify the formation of mitochondrial ROS (Figure 3A). The MitoSOX Green reagent is chemically targeted to mitochondria for highly selective detection of superoxide of live cells and exhibits green fluorescence. Confocal microscopic imaging demonstrated a prominent enhancement in green fluorescence signal in differentiated PC12 cells treated with 100 μM NMDA for 24 h, indicating an increase in superoxide levels in mitochondria. The NMDA-induced increase in mitochondrial superoxide was partly prevented when CHPG was added, as indicated by a blunting of the MitoSOX fluorescent signal. Consistent with this finding, MitoSOX analysis by flow cytometry revealed a significant increase in mean intensity of fluorescence in cells treated with NMDA, and a decrease in cells pretreated with CHPG (Figure 3B). Moreover, the MMP value was significantly lower than the control value after exposure to 100 μM NMDA for 24 h, and increased toward depolarization after pretreatement with CHPG (Figure 3C). To assess whether CHPG could induce the release of cytochrome c from mitochondria, cytochrome c levels in mitochondria and cytosol were measured via immunoblotting. As shown in Figure 3D,E, cells exposed to 100 μM NMDA showed a significant decrease in the expression of cytochrome c in mitochondria and a concomitant increase in the cytosolcytosol. However, this cytochrome c release was significantly attenuated when cells were pretreated with CHPG.

### 2.4. CHPG Disrupts the Formation of NMDAR-PSD-95 Complex

**Figure 4:**
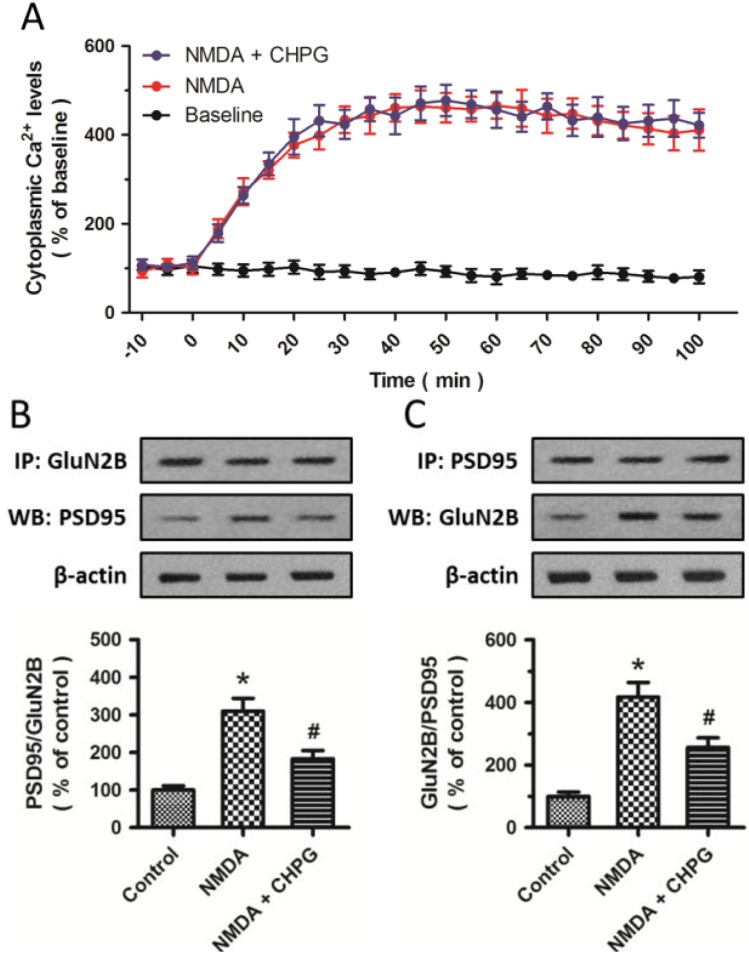
CHPG disrupts the formation of NMDAR-PSD-95 complex. Differentiated PC12 cells were treated with 100 μM NMDA alone or pretreated with 50 μM CHPG before NMDA treatment. (**A**) Cytoplasmic Ca^2+^ levels were measured by calcium imaging up to 100 min after injury; (**B**,**C**) Coimmunoprecipitation experiments showed the effects of CHPG on NMDAR-PSD-95 interaction. Samples were immunoprecipitated and analyzed by western blotting with the indicated antibodies and the data was analyzed. Data represent means ± SD from five experiments. * *p* < 0.05 *vs.* control group and ^#^ *p* < 0.05 *vs.* cells treated only with NMDA.

To assess the effects of CHPG on changes in intracellular Ca^2+^ concentration after NMDA insults, cytoplasmic Ca^2+^ levels were measured by calcium imaging (Figure 4A). NMDA treatment significantly increased the intracellular Ca^2+^ concentration, suggesting that excitotoxicity was partially dependent on the disturbance of intracellular Ca^2+^ homeostasis, which was consistent with previous studies [14,15]. However, further study showed that pretreatment with CHPG did not influence this effect, suggesting that the protective effect of CHPG on NMDA-induced neurotoxicity was not dependent on the alteration of intracellular Ca^2+^ concentration. To further explore whether mGluR5 activation disrupts NMDA-induced NMDAR-PSD-95 interaction, coimmunoprecipitation experiments of PSD-95 with the GluN2B subunit were used to examine total GluN2B-PSD-95 complex levels in differentiated PC12 cells. The results showed that NMDA significantly increased the coimmunoprecipitation of PSD-95 with GluN2B, while CHPG could reduce this increase (Figure 4B,C). Thus, pretreatment with CHPG significantly inhibited the NMDA-induced increase in GluN2B-PSD-95 complex formation.

### 2.5. Discussion

Here, we demonstrate that NMDA alone induces dose-dependent neurotoxicity, and activation of mGluR5 by using the selective agonist CHPG attenuates NMDA-induced cell death in differentiated rat PC12 cells. Moreover, CHPG significantly reduces the number of cells with apoptotic morphology after exposure to NMDA, which is associated with down-regulated expression of caspase-9, caspase-3, and Bcl-2/Bax ratio. Besides, CHPG also decreases mitochondrial cytochrome c release, which is widely accepted as the critical step for the formation of the apoptosome. Lastly, although CHPG has negative effects on the changes of intracellular Ca^2+^ concentration induced by NMDA treatment, the NMDAR-PSD-95 complex is impacted by CHPG, supporting the concept that NMDAR-PSD-95 complex contributes to NMDA-induced cell death.

Of the mGluRs family, group I mGluRs (type 1 and type 5) have gained the most attention. Multiple studies have shown that mGluR5 plays a key role in the modulation of diverse cellular responses [16,17,18]. It has long been known that the distribution of postsynaptic mGluR5 is at perisynaptic or extrasynaptic sites, and therefore will sense relatively low concentrations of glutamate diffusing out of the synaptic cleft [19]. However, it has been reported that selective blocking of mGluR5 does not provide neuroprotection other than blocking NMDAR [20,21]. In contrast, activation of mGluR5 has been shown to be neuroprotective in cell culture models of etoposide or staurosporine-induced neuronal apoptosis [22], which was shown to be highly caspase-3 dependent [23]. In addition, mGluR5 may participate in the events underlying long-term depression (LTD), a form of synaptic plasticity that likely contributes to learning and memory [24], induced by electrical stimulation of the Schaffer collateral pathway [17]. Consistent with those findings, our previous studies also have provided evidence that activation of mGluR5 with CHPG has anti-apoptotic and neuroprotective effects in *in vitro* and *in vivo* traumatic brain injury (TBI) models [25]. The possible mechanisms through which CHPG provides protective effects might involve the PKC-dependent activation of the MER/ERK pathway [26]. However, our present findings suggest that mGluR5 may modulate cell apoptosis not only through the pathways mentioned above, but also other signaling cascades that lead to cell death.

According to our research, one of the potential underlying mechanisms of the CHPG-induced protective effects might be disruption of the NMDAR-PSD-95 complex, which in turn promotes cell survival. It is increasingly evident that PSD-95 plays an important role in apoptotic pathways in diseases involving excitotoxicity and ischemic brain damage [27,28]. At excitatory synapses, PSD-95 functions as a scaffolding protein, binding both NMDARs and nNOS through its second postsynaptic density-95⁄Dlg⁄ZO1(PDZ) domain [29], assembling them into multiprotein signaling complexes, and is subsequently involved in diverse functions, such as signal transduction, structural regulation, and metabolism [27]. However, neither genetically disrupting PSD-95 *in vivo* [30], nor suppressing its expression *in vitro* [31] can affect NMDAR-dependent excitotoxicity. Growing evidence supports the fact that proper targeting of interactions between PSD-95 and proteins downstream is essential for its regulation of NMDAR function [10,29], which plays an essential role in mediating ischemic brain injury and stroke. Therefore, targeting the PSD-95 protein by disrupting its interaction with other proteins, such as nNOS and NMDARs, might be an alternative strategy for treating multiple brain disorders. It has been reported that blocking the nNOS translocation from the cytosol to the membrane, which is provoked by NMDAR activation, by dissociating nNOS and PSD-95 ameliorates cerebral ischemic injury [32]. Our results demonstrate that CHPG could disrupt the increased formation of the NMDAR-PSD-95 complex in NMDA-induced neurotoxicity, which is consistent with recent reports showing that interfering with the interaction between NMDARs and PSD-95 may interrupt signaling that leads to neuronal death downstream from NMDARs [10].

Numerous previous studies have demonstrated a positive coupling between group I mGluRs and NMDARs [33,34]. The positive mGluR5-NMDAR interaction plays an important role in normal glutamatergic transmission and synaptic plasticity, which is implicated in the pathogenesis of a variety of neurological disorders, including pain, addiction, neurodegenerative diseases, and neurotoxicity [35]. The group I mGluRs-induced regulation of NMDARs is thought to act through a post-synaptic mechanism [36]. Although mGluR5 has no direct binding site for NMDAR or PSD-95, it is possible that mGluR5 attenuates NMDA-induced neurotoxicity by facilitating the signaling from mGluRs to some other proteins, namely, Homer proteins [37], which are reported to have direct interaction with mGluRs [38]. Homer then binds to Shank, a scaffold protein lying in the deep part of PSD-95, to form a polymeric network structure [39]. The postsynaptic complex formed by Homer and Shank proteins after mGluR5 activation might block the interaction between PSD-95 and NMDAR, and attenuates the excitotoxicity induced by NMDA treatment, which needs to be further determined.

Intracellular Ca^2+^ mobilization in neuronal cells is crucial for NMDA-induced neurotoxicity [40]. The disturbance in the intracellular Ca^2+^ level is one of the critical initiators of NMDA-induced neurotoxicity and apoptosis. Although the changes in intracellular Ca^2+^ levels are triggered by the activation of plasma membrane channels, like NMDAR or mGluRs, the consequent increase in free cytosolic Ca^2+^ also can be modulated by the activity of intracellular calcium stores [41,42,43]. As major Ca^2+^ regulating organelles, the endoplasmic reticulum and mitochondria play an essential role in modulating and interpreting Ca^2+^ signals, taking part in Ca^2+^ uptake, sequestration, and release [44]. Mitochondrial calcium handling attracts special interest and plays a pivotal role in Ca^2+^ signaling in neurons. Besides, calcium overload and subsequent dysfunction of mitochondria are thought to be critically essential for initiating the cell death following TBI [45], ischemia [46], and other neurodegenerative disorders [47,48]. However, the underlying mechanisms of mitochondrial dysfunction in neurotoxicity still remain unclear. Anti-exicitotoxic therapies targeted at glutamate receptors have failed to protect against ischemic injuries [49], indicating that NMDAR is the main, but not necessary only, pathway for Ca^2+^ entry. Interestingly, in our study, although the activation of mGluR5 by CHPG might inhibit the activation of the mitochondria-associated apoptotic pathway, it has no effects on NMDA-related changes in Ca^2+^ concentration in excitotoxicity. All these data indicated an intracellular calcium independent neuroprotective mechanism against NMDA-induced neuronal injury induced by the mGluR5 agonist CHPG.

## 3. Experimental Section

### 3.1. Chemicals and Reagents

Antibodies against PSD-95, Bax, Bcl-2, caspase-9, cleaved caspase-9, and cleaved caspase-3 were obtained from Cell Signaling Technology (Danvers, MA, USA). The antibody against GluN2B was obtained from NeuroMab (Davis, CA, USA). CHPG, MPEP, NMDA, and the antibody against β-actin was obtained from Sigma (St Louis, MO, USA). The secondary antibodies for immunostaining were Alexa 488 donkey anti-(goat Ig) and Alexa 594 donkey anti-(rabbit Ig) (Invitrogen, CA, USA). The secondary antibodies for immunoblotting were horseradish peroxidase-conjugated anti-rabbit, anti-mouse and anti-goat IgG (Santa Cruz Biotechnology, Santa Cruz, CA, USA). CHPG and NMDA were dissolved in saline. Cells were pretreated with CHPG for 1h before 24 h NMDA treatment to induce excitotoxicity.

### 3.2. Cell Culture

PC 12 cells were obtained from the Institute of Biochemistry and Cell Biology, SIBS, CAS, and plated onto poly-l-lysine-coated 40 mm glass coverslips or glass culture dishes at a density of 5000 cells/cm^2^. Cell were grown in Dulbecco’s modified Eagle’s medium (DMEM) supplemented with 10% fetal bovine serum and 100 units/mL penicillin/streptomycin. The cells were incubated in a humidified atmosphere (95% air and 5% CO_2_) at 37 °C. To mimic neuronal injury *in vitro*, cells were induced to differentiate with 50 ng/mL nerve growth factor (NGF) for 48 h, as previously published [50]. Before use, cells were maintained for at least 7 days in the NGF-containing medium, which was changed every 48 h.

### 3.3. Cell Viability Assay

Cell viability assay was performed with The Cell Proliferation Reagent WST-1 (Roche, Basel, Switzerland), following the manufacturer’s instructions. PC12 Cells were cultured (at a concentration of 0.5 × 10^4^–5 × 10^4^) in microplates (tissue culture grade, 96 wells, flat-bottomed) in a final culture medium volume of 100 μL/well. After various experimental treatments, 10 μL Cell Proliferation Reagent WST-1 was added into each well and incubated for 4 h at 37 °C and 5% CO_2_. The absorbance of 100 μL culture medium plus 10 μL WST-1 in the absence of cells in one well was used as background control. Microplates then were shook thoroughly for 1 min on a shaker, and the absorbance of the samples was measured against a background control as a blank with the ELISA reader. Cell viability was measured as a percentage of the values of cells without any treatment.

### 3.4. LDH (Lactate Dehydrogenase) Release Assay

Cell cytotoxicity was determined by the LDH release assay, the smallest cytoplasmic enzyme released from cells, and a marker of membrane integrity. The amount of LDH release into the culture medium was detected using a diagnostic kit (Jiancheng Bioengineering Institute, Nanjing, China), according to the manufacturer’s protocol. Briefly, the samples collected from each well were incubated with nicotinamide-adenine dinucleotide (NADH) and pyruvate for 15 min at 37 °C. Then the reaction was stopped with 0.4 mol NaOH. Relative absorbance was measured at 490 nm to determine the activity of LDH, and background absorbance from culture medium, determined in intact control cultures, was subtracted from all absorbance values. The results were normalized to the maximal LDH release, which was determined by treating control cultures for 60 min with 1% Triton X-100 to lyse all cells.

### 3.5. Flow Cytometry

Differentiated PC12 cells were seeded in 6-well plates at a density of 5 × 10^5^/well, and harvested 24 h after treatment with NMDA as described. Cells were washed twice with ice-cold PBS and re-suspended in binding buffer. The cell suspension was transferred into a tube and double-stained with Annexin V-FITC and propidium iodide (PI) at room temperature in the dark for 15 min, according to the Annexin V apoptosis detection kit instructions. The percentage of apoptotic cells was quantified by flow cytometry at 530 and 600 nm to measure green Annexin V-FITC and red PI fluorescence respectively (FACSCalibur, BD Biosciences, San Jose, CA, USA). Further differentiation between apoptotic *vs.* necrotic types of cell death was studied by Annexin V and PI staining in different quadrants: Q1 (AV^−^/PI^+^, the necrotic cells), Q2 (AV^+^/PI^+^, the late phase apoptotic cells), Q3 (AV^−^/PI^−^, normal cells) and Q4 (AV^+^/PI^−^, the early phase apoptotic cells).

### 3.6. Immunostaining

To assess the activation of caspase-3 in the differentiated rat PC12 cells , cells were fixed with 4% paraformaldehyde for 15 min at room temperature, and then cells were washed with NaCl/P_i_ and permeabilized with 0.2% Triton X-100, which was followed by incubation overnight at 4 °C with anti-cleaved caspase-3 (1:500). Cells were then exposed to secondary antibodies [Alexa 594-conjugated goat (anti-mouse Ig), 1:300] for 2 h, washed, dehydrated with ethanol, and stained with 4,6-diamidino-2-phenylindole (DAPI, Sigma). Images were captured using an Olympus FV10i Confocal Microscope (Tokyo, Japan). All images of one experiment were acquired in the same exposure time, allowing comparisons of relative levels of immunoreactivity between the different treatment conditions.

### 3.7. MitoSOX Assay

Mitochondrial superoxide production was measured by using the MitoSOX Red Kit (Invitrogen, Carlsbad, CA, USA). Briefly, cells were incubated with MitoSOX Red for 10 min in a CO_2_ incubator at 37 °C. MitoSOX was added to the medium to a final concentration of 5 μM after treatment and cells were rinsed with the perfusion buffer before imaging. Following incubation, MitoSOX Red fluorescence intensity was acquired at 510/580 nm on an Olympus FV10i Confocal Microscopy equipped with a digital cooled charged-coupled device camera.

### 3.8. Measurement of Mitochondrial Membrane Potential (MMP)

MMP was measured using the fluorescent probe JC-1 as described previously [51]. After various treatments, cells were incubated with JC-1 in basic medium for 15 min at 37 °C in the dark. After two more rinses with Hank’s solution, coverslips were viewed on an Olympus Optical (Tokyo, Japan) IX70 inverted fluorescence microscope. Relative fluorescence intensities were monitored at an excitation of 490 nm and emission of 530 nm (green fluorescent monomers) and 590 nm (red fluorescent aggregates), respectively. The numbers of mitochondria stained red-orange or green cells were counted, and the degree of mitochondrial depolarization was measured as the percentage of green cells per field.

### 3.9. Quantification of Cytochrome C Release

The cytochrome c levels of cells in response to different treatments were measured using the Quantikine M Rat/Mouse Cytochrome C Immunoassay kit (R&D Systems, St. Paul, MN, USA). Briefly, cultured PC12 cells were harvested and lysed with a lysis buffer containing protease inhibitors. The protein content in each fraction was estimated using a BCA protein assay kit (Jiancheng Bioengineering Institute, Nanjing, China). The cell lysate was centrifuged to remove pellets containing the nuclei and intact cells. The supernatant was then centrifuged at 15,000× *g* for 15 min. The pellet fraction containing mitochondria was further incubated with PBS containing 0.5% Triton X-100 for 10 min. After centrifugation, the resulting supernatant was identified as the cytosolic fraction, and the pellet was identified as mitochondrial fraction. All steps were performed on ice or 4 °C. The cytochrome c concentration was expressed as ng/mg protein.

### 3.10. Calcium Imaging

Intracellular Ca^2+^ concentrations were measured with the ratiometric Ca^2+^ indicator Fluo3-AM (Invitrogen, Sacramento, CA, USA). Cultured cells grown on glass slides were loaded with 5 μM Fluo3-AM for 30–40 min before NMDA treatment at room temperature. Cells were then placed in an open-bath imaging chamber containing Dulbecco’s PBS (0.901 mM CaCl_2_, 0.493 mM MgCl_2_·6H2O, 2.67 mM KCl, 1.47 mM KH_2_PO_4_, 137.93 mM NaCl, 8.06 mM Na_2_HPO_4_·7H_2_O, pH 7.2–7.4) supplemented with 100 μM NMDA at ambient temperature. The cells had an almost homogenous resting fluorescence after loading with Fluo3-AM. Fluo3 was excited at 488 nm with an argon ion laser on a Nikon inverted epifluorescence microscope, and the emission fluorescence at 510 nm was acquired. Images were recorded and analyzed with Meta Flour image-processing software. Post analysis of the Ca^2+^ concentration values was calculated with Ca^2+^-insensitive fluorescence subtracted from each wavelength.

### 3.11. Coimmunoprecipitation

Coimmunoprecipitation was performed by following a previously published procedure [8]. Briefly, cells either untreated or treated with CHPG were harvested in a PBS-based buffer containing 1 mM EGTA, 1 mM EDTA, 1 mM PMSF, 2 μg/mL aprotinin, 20 μg/mL pepstatin A, and 20 μg/mL leupeptin. After centrifugation, cells were lysed and solubilized in harvesting buffer containing 0.1% SDS and 0.8% Triton X-100. An equal amount of the lysate (one-tenth) was reserved for input loading, and the remainder was incubated with protein-A/G beads for 1 h at 4 °C to remove any nonspecifically bound proteins. The supernatant was collected and centrifuged, and then incubated with a rabbit antibody against GluN2B for 1 h at 4 °C. The complex was precipitated with 50% protein A/G beads slurry. Proteins were separated and detected on immunoblots with antibodies against GluN2B subunit and PSD-95. The amount of PSD-95 Co-IP with GluN2B was measured as the PSD-95 to GluN2B, or GluN2B to PSD-95 band-density ratio.

### 3.12. Western Blotting

PC12 cells were seeded into 6-well plates. After various treatments, total protein was extracted for western blotting. The cells were washed three times with ice-cold NaCl/Pi, and then lysed in lysis buffer containing protease and phosphatase inhibitor mixture tablets (PhosSTOP; Roche). The total proteins were separated in 10%–15% SDS-PAGE gels and transferred onto nitrocellulose membranes (Invitrogen). The membranes were blocked with 5% skim milk solution diluted in NaCl/Tris with 0.05% Tween-20 (NaCl/Tris-T) for 1 h at room temperature. After blocking, the membranes were incubated with antibodies (anti-Bax Ig, 1:1000; anti-Bcl-2 Ig, 1:1000; anticaspase-9 Ig, 1:800; anti-cleaved caspase-9 Ig, 1:800; anti-PSD-95 Ig, 1:800; anti-GluN2B Ig, 1:500; anti-b-actin Ig, 1:2500) overnight at 4 °C. The membranes were washed three times with NaCl/Tris-T, and then incubated with the secondary antibodies for 1 h at room temperature. Protein bands were detected with SuperSignal West Pico Chemiluminescent Substrate (Thermo Scientific, Rockford, IL, USA). The absorbance values of the bands were quantified using an image analysis system and IMAGEJ 1.48 (National Institutes of Health, Boston, MA, USA). The protein levels were normalized against β-actin intensity.

### 3.13. Statistical Analysis

Statistical evaluation was performed with GraphPad Prism Program, version 5.0 (La Jolla, CA, USA). Quantitative values were presented as means ± standard error of the mean (SEM). Protein expression by western immunoblot was tested using a student’s *t*-test. Differences between experiments were assessed by univariate one-way analysis of variance (ANOVA) (more than two groups) followed by Bonferroni’s multiple comparisons or unpaired *t*-tests (two groups). A value of *p* < 0.05 was considered statistically significant.

## 4. Conclusions

In conclusion, our study provides evidence that activation of mGluR5 reduces NMDA-induced excitotoxic neuronal injury by disrupting the formation of the NMDAR-PSD-95 complex and inhibiting the mitochondrial-associated apoptotic pathway. These results demonstrate that mGluR5 might act competitively against ionotropic glutamate receptors to integrate with PSD-95 indirectly through proteins downstream, such as Homer and Shank, mediating the functions of mitochondria. Therefore, pharmacological activation of mGluR5 would provide an alternative therapeutic strategy for preventing and treating excitotoxicity-related neurological diseases.
